# p300 Serine 89: A Critical Signaling Integrator and Its Effects on Intestinal Homeostasis and Repair

**DOI:** 10.3390/cancers13061288

**Published:** 2021-03-14

**Authors:** Keane K. Y. Lai, Xiaohui Hu, Keisuke Chosa, Cu Nguyen, David P. Lin, Keith K. Lai, Nobuo Kato, Yusuke Higuchi, Sarah K. Highlander, Elizabeth Melendez, Yoshihiro Eriguchi, Patrick T. Fueger, Andre J. Ouellette, Nyam-Osor Chimge, Masaya Ono, Michael Kahn

**Affiliations:** 1 Department of Molecular Medicine, Beckman Research Institute of City of Hope, Duarte, CA 91010, USA; klai@coh.org (K.K.Y.L.); huxiaohui@ahmu.edu.cn (X.H.); kchosa@coh.org (K.C.); cunguyen@coh.org (C.N.); dplin@hotmail.com (D.P.L.); yhiguchi@coh.org (Y.H.); emelendez@coh.org (E.M.); nchimge@coh.org (N.-O.C.); 2 City of Hope Comprehensive Cancer Center, Duarte, CA 91010, USA; pfueger@coh.org; 3 Department of Anatomic Pathology, Cleveland Clinic, Cleveland, OH 44195, USA; LAIK2@ccf.org; 4 The Institute of Scientific and Industrial Research, Osaka University, Osaka 567-0047, Japan; kato-n@sanken.osaka-u.ac.jp; 5 Clinical Microbiome Service Center and Pathogen and Microbiome Division, Translational Genomics Research Institute, Flagstaff, AZ 86005, USA; shighlander@tgen.org; 6 Department of Pathology and Laboratory Medicine, Keck School of Medicine, University of Southern California, Los Angeles, CA 90033, USA; eriguchi@intmed1.med.kyushu-u.ac.jp (Y.E.); aouellet@med.usc.edu (A.J.O.); 7 Department of Molecular and Cellular Endocrinology, Beckman Research Institute of City of Hope, Duarte, CA 91010, USA; 8 USC Norris Comprehensive Cancer Center, Keck School of Medicine, University of Southern California, Los Angeles, CA 90033, USA; 9 Department of Clinical Proteomics, National Cancer Center Research Institute, Tokyo 104-0045, Japan; masono@ncc.go.jp; 10 Department of Biochemistry and Molecular Biology, Keck School of Medicine, University of Southern California, Los Angeles, CA 90033, USA

**Keywords:** CBP, p300, IBD, colitis, colorectal cancer, Wnt

## Abstract

**Simple Summary:**

Given their high degree of identity and even greater similarity at the amino acid level, Kat3 coactivators, CBP (Kat3A) and p300 (Kat3B), have long been considered redundant. We describe the generation of novel p300 S89A knock-in mice carrying a single site directed amino acid mutation in p300, changing the highly evolutionarily conserved serine 89 to alanine, thus enhancing Wnt/CBP/catenin signaling (at the expense of Wnt/p300/catenin signaling). p300 S89A knock-in mice exhibit multiple organ system, immunologic and metabolic differences, compared with their wild type counterparts. In particular, these p300 S89A knock-in mice are highly sensitive to intestinal injury resulting in colitis which is known to significantly predispose to colorectal cancer. Our results highlight the critical role of this region in p300 as a signaling nexus and provide further evidence that p300 and CBP are non-redundant, playing definite and distinctive roles in development and disease.

**Abstract:**

Differential usage of Kat3 coactivators, CBP and p300, by β-catenin is a fundamental regulatory mechanism in stem cell maintenance and initiation of differentiation and repair. Based upon our earlier pharmacologic studies, p300 serine 89 (S89) is critical for controlling differential coactivator usage by β-catenin via post-translational phosphorylation in stem/progenitor populations, and appears to be a target for a number of kinase cascades. To further investigate mechanisms of signal integration effected by this domain, we generated p300 S89A knock-in mice. We show that S89A mice are extremely sensitive to intestinal insult resulting in colitis, which is known to significantly increase the risk of developing colorectal cancer. We demonstrate cell intrinsic differences, and microbiome compositional differences and differential immune responses, in intestine of S89A versus wild type mice. Genomic and proteomic analyses reveal pathway differences, including lipid metabolism, oxidative stress response, mitochondrial function and oxidative phosphorylation. The diverse effects on fundamental processes including epithelial differentiation, metabolism, immune response and microbiome colonization, all brought about by a single amino acid modification S89A, highlights the critical role of this region in p300 as a signaling nexus and the rationale for conservation of this residue and surrounding region for hundreds of million years of vertebrate evolution.

## 1. Introduction

**Figure 1:**
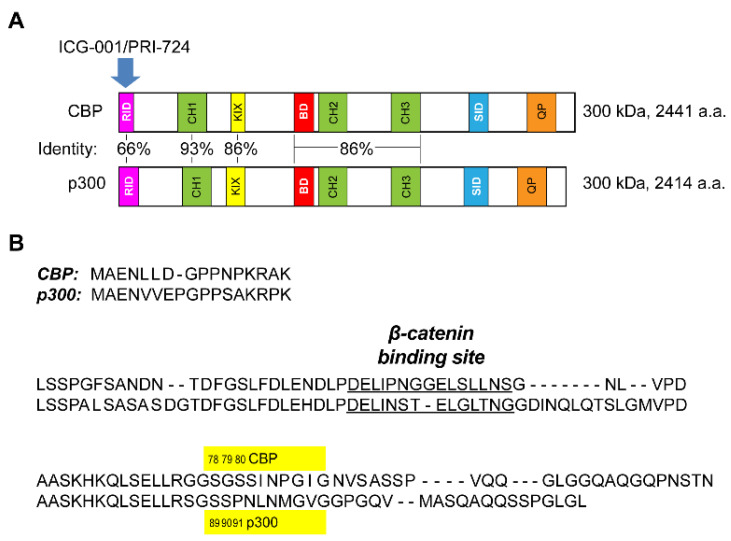
Differential usage of homologous Kat3 coactivators CBP and p300 by β-catenin. (**A**) Schematic representation displaying identity between CBP and p300. CBP and p300 have molecular weights of approximately 300 kDa and are encoded over 33 and 31 exons and consist of 2441 and 2414 amino acids (a.a.), respectively. β-catenin competes with direct small molecule CBP/catenin antagonists (PRI-724/ICG-001) for binding to CBP’s (but not p300′s) distal N-terminus, the least conserved region within these two Kat3 coactivators. CBP, cAMP response element binding protein (CREB)-binding protein; p300, E1A-binding protein, 300 kDa; RID, receptor-interacting domain; CH, cysteine/histidine region; KIX, kinase-inducible domain interacting domain; BD, bromodomain; SID, steroid receptor co-activator-1 interaction domain; QP, glutamine- and proline-rich domain. (**B**) Sequence alignment of the distal N-terminal regions of CBP and p300, showing conserved sites for binding of β-catenin (DELI motif). Note: p300 S89 is a critical residue controlling differential Kat3 coactivator usage by β-catenin. (Human CBP and p300 sequences are depicted.)

The vertebrate radiation, which was initiated approximately 450 million years ago, ushered in a major lifestyle change with a significant increase in adult lifespan [1,2] and with it, a requirement for high-fidelity, long-term homeostasis [2]. This change necessitated that somatic stem cells (SSC), in their respective niches, remain quiescent, in contrast to their differentiated daughter cells, which rapidly proliferate, in order to safeguard the integrity of the SSC’s genetic material [1,3]. The gene duplication of the Kat3 coactivator family, which led to the evolution of Kat3A/CREBBP (cAMP response element binding protein (CREB)-binding protein) (CBP) and its closely related paralog Kat3B/E1A-binding protein, 300 kDa (p300), apparently occurred just prior to radiation of the vertebrate lineage [1]. The two Kat3 coactivators encode massive proteins of ~300 kDa over 33 and 31 exons, respectively [1]. CBP and p300 have maintained an extraordinarily high degree of identity—as high as 93%—and an even higher degree of similarity, particularly over an extensive central core region, which encompasses the CH1, KIX, bromodomain, CH2 and CH3 domains (Figure 1A) [1,2,4,5]. They both interact with a myriad of proteins, given their key roles in orchestrating transcription [1]. Due to their high degree of identity and even greater similarity at the amino acid level, CBP and p300 have long been considered redundant. However, mounting evidence clearly demonstrates that they are non-redundant, playing definite and distinctive roles in development and disease [1,6,7,8,9,10]. β-catenin, a key transcriptional component in Wnt signaling, must recruit CBP or p300 in addition to other components of the core transcriptional complex to initiate functional Wnt transcription [1,11,12]. The extreme N-terminal regions of CBP and p300, containing the lowest homology with approximately 66% identity, have been the focus of our interest. β-catenin and specific, direct small molecule CBP/catenin antagonists (ICG-001/PRI-724) [1,9,13,14] and direct small molecule p300/catenin antagonists (YH249/250) [15], competitively bind within this extreme N-terminal region (Figure 1A) [1]. This highly unstructured region of the Kat3 coactivators serves as a nexus for integrating the interactions of varied signal transduction pathways (e.g., nuclear receptor family, RAR/RXR, vitamin D, and Interferon STAT1/2) with the Wnt/catenin signaling cascade [1,9,16,17,18,19]. We originally identified p300 serine 89 as a critical residue (Figure 1B) controlling differential coactivator usage by β-catenin via post-translational phosphorylation in mouse embryonic stem cells [20]. p300 serine 89 appears to be a target for a number of kinase cascades including PKC [21,22], AMPK [23], and SIK2 [24], associated with an array of biological effects, including activation and inhibition of transcription [23,25], inhibition of histone acetyltransferase function [22], regulation of insulin/glucagon signaling [24], and differentiation of mES cells [20] and adult progenitor cells [26]. 

To further investigate the role that p300 serine 89 (S89) plays in vivo, we have generated p300 S89A knock-in mice. p300 S89A knock-in mice, albeit born at sub-Mendelian ratios, appear to be relatively normal. Nevertheless, these mice exhibit multiple organ system, immunologic and metabolic differences, compared with their wild type counterparts. We now initially report on the generation of these mice and their high sensitivity to intestinal injury, which is apparently related to a complex interplay between aberrant epithelial differentiation, gut immunity and changes in their intestinal microbiota and metabolites and which results in colitis, a significant risk factor predisposing to colorectal cancer [27,28].

## 2. Materials and Methods

### 2.1. Mice

Animal studies were approved by the University of Southern California Institutional Animal Care and Use Committee (IACUC) as per protocol #11023. The S89A knock-in point mutation in exon 2 of the mouse p300 gene, via site-specific mutagenesis, was generated using the flip-excision (FLEx) switch construct. This mutation removes the highly conserved phosphorylation site at S89. The construct included five segments: the 5′ homology arm, a point-mutated exon 2 in inverted orientation, a PGK-Neo selection cassette, the wild type exon 2, and the 3′ homology arm. The design of the construct, cloning of the targeting vector, electroporation into mouse ES cells, screening of the 129 ES cells, injection into blastocysts, and screening of the chimeric mice were performed by Ozgene Pty Ltd. (Australia). In principle, transcription from the mutant exon 2 should have been activated via Cre recombinase in two steps. First, Cre recombinase would invert the mutated fragment flanked by the *loxP* site to correct the orientation to activate, and then excise the wild type fragment flanked by lox2272 to inactivate it. However, we found that mice homozygous for the knock-in construct displayed early embryonic stage lethality, similar to that of p300 knockout mice [29], suggesting that the wild type p300 protein was not being produced properly from the wild type fragment in vivo for unknown reasons. This malfunction of the wild type fragment caused us to revise our original plan of conditional mutagenesis, resulting in our decision to generate p300 S89A germ line mice. Mice were backcrossed onto C57BL/6 background (The Jackson Laboratory, Bar Harbor, ME, USA) for at least 10 generations before used for experiments. Hematology testing on mouse blood samples was performed on the Hemavet (Drew Scientific, Miami Lakes, FL, USA), and clinical chemistry testing was performed by Antech Diagnostics. 

### 2.2. Isolation of Crypt Cells from Ileum

Crypt cells from ileum were isolated based on a previously described protocol [30] with minor modification. Ileum was dissected out, and lumen of the intestine was flushed with ice-cold PBS. Intestine was opened longitudinally and placed in tube containing ice-cold PBS. Tube was inverted 10–15 times, and then PBS removed and replaced with fresh ice-cold PBS. Washing with fresh ice-cold PBS was repeated until the supernatant no longer contained any visible debris. Intestine was cut into 5 mm pieces and placed into ice-cold 5 mM EDTA-PBS. Fragments of intestine were vigorously triturated by pipetting up and down 15 times, and then allowed to settle by gravity for 30 s. Supernatant was aspirated, and then 5 mM EDTA-PBS was added to the intestinal fragments and re-suspended intestinal fragments were placed at 4 °C on a benchtop roller for 10 min, after which supernatant was aspirated, and then intestinal fragments kept. 5 mM EDTA-PBS was added to the intestinal fragments and then placed at 4 °C on a benchtop roller for 30 min. Supernatant was aspirated and then ice-cold PBS was added to wash the crypts and then supernatant was aspirated. Ice-cold PBS was added, and the intestinal fragments were vigorously triturated 10 times. Supernatant fractions were collected and then mixed 1:1 with solution of basal media containing DNase I. (Final concentration of mixture: ~15 U/mL DNase I.) Mixture was first filtered through a 100 μm filter into a BSA (1%) coated conical tube, and then filtered through a 70 μm filter into a BSA (1%) coated tube, after which the filtrate was spun at 300× *g* in a tabletop centrifuge for 5 min. Supernatant was aspirated, and the cell pellet was re-suspended in basal media containing 5% FBS and then centrifuged at 100× *g* for 5 min, after which supernatant was removed and samples were frozen at −80 °C until further analysis.

### 2.3. Co-Immunoprecipitation 

150–200 mg of intestinal crypt cells were re-suspended in CERI buffer (NE-PER, ThermoFisher, cat. #: 78833, Waltham, MA, USA) containing 5 mM DTT and 1X protease inhibitor cocktail, using a dounce homogenizer. After re-suspension in CERI buffer, the procedure for nuclear extraction was performed based on manufacturer protocol. Protein concentration of nuclear extract was performed using the Protein Assay Dye Reagent (Bio-Rad, cat. #: 500-0006, Hercules, CA, USA). 100–500 µg of nuclear protein was diluted in CoIP buffer (25 mM Tris-HCl, pH 8.0, 1% NP40, 5% glycerol, 150 mM NaCl, 1 mM EDTA, 5 mM DTT, 1X protease inhibitor cocktail (Calbiochem, cat. #: 539137, Burlington, MA, USA)) to a final volume of 1000 µL. 2 µg of CBP (Aviva Biosystems, cat. #: ARP43609_P050, San Diego, CA, USA), p300 (Aviva Biosystems, cat. #: OAAF01891-100UG, San Diego, CA, USA), 14-3-3-Ɛ (Abcam, cat. #: ab43057, Cambridge, MA, USA), or normal IgG (Aviva Biosystems, cat. #: OAEF01185-1MG, San Diego, CA, USA) antibody was added and mixture incubated overnight at 4 °C on a tube shaker/rotator. 20 µL of Dynabeads Protein A (ThermoFisher, cat. #: 10001D, Waltham, MA, USA) was added and mixture incubated for 1 h at 4 °C on tube shaker/rotator. The magnetic beads were washed three times with 500 µL of CoIP Buffer each time, using the magnetic stand. 20 µL of 2× Laemmli buffer was added and mixture vortexed. Beads were boiled in 2× Laemmli buffer for 10 min. Supernatant containing proteins were separated from the magnetic beads, using the magnetic stand. Protein samples were subjected to electrophoresis on a 4–20% Teo-Tricine SDS-PAGE gel (VWR, cat. #: 71003-072, Radnor, PA, USA). Proteins were transferred onto PVDF membrane. Proteins of interest on the PVDF membrane were detected by incubating with β-catenin antibody (Santa Cruz Biotechnology, cat. #: sc-7199, Dallas, TX, USA) or p300 antibody (Aviva Biosystems, cat. #: OAAF01891-100UG, San Diego, CA, USA) as the primary antibody, and subsequent incubation with CleanBlot (ThermoFisher, cat. #: 21232, Waltham, MA, USA) as the secondary antibody, followed by application of chemiluminescent reagent ECL Plus (GE Healthcare, cat. #: RPN2132, Chicago, IL, USA) and imaging on the ChemiDoc Imaging System (Bio-Rad), after which relative protein concentration was determined by densitometry.

### 2.4. Western Blotting 

Flash-frozen mouse ileum was resuspended in RIPA buffer (50 mM Tris-HCl, pH 7.5, 150 mM NaCl, 1% NP-40, 0.5% deoxycholate, 5 mM EDTA, 0.1% SDS), containing protease inhibitors (Roche, cat. #: 11836170001, South San Francisco, CA, USA). Tissue was homogenized and then centrifuged at 12,000× *g* for 15 min, after which supernatants were collected. Protein concentration was determined using the Protein Assay Dye Reagent (Bio-Rad, cat. #: 500-0006, Hercules, CA, USA). 40 µg protein mixed with 4× Laemmli sample buffer were incubated at 37 °C for 15 min and subjected to SDS-PAGE. After overnight transfer onto PVDF membrane (Bio-Rad, cat. #: 1620177, Hercules, CA, USA), membrane was incubated overnight with primary antibody at 4 °C. Membrane was washed and then incubated with secondary antibody for 1 h, followed by application of chemiluminescent reagent ECL (GE Healthcare, cat. #: RPN2232, Chicago, IL, USA) and imaging on the ChemiDoc Imaging System (Bio-Rad), after which relative protein concentration was determined by densitometry. The primary antibodies used were DUOX2 (Santa Cruz, cat. #: sc-398681, Dallas, TX, USA), GAPDH (Santa Cruz, cat. #: sc-32233, Dallas, TX, USA). The secondary antibody was mouse IgG kappa binding protein conjugated to horseradish peroxidase (Santa Cruz, cat. #: sc-516102, Dallas, TX, USA).

### 2.5. RT-qPCR and PCR 

Total mRNA was extracted by TRIzol reagent (Invitrogen Carlsbad, CA, USA) according to the manufacturer’s protocol. cDNA was synthesized using qScript cDNA Synthesis Kit (Quantabio, Beverly, MA, USA). cDNA template was used for qPCR with SYBR Green detection method utilizing the CFX Connect Real-Time PCR Detection System (Bio-Rad, Hercules, CA, USA). The PCR primer sequences used for mouse cells were as follows: Duox2 (F: 5′-CATTGCCACCTACCAGAACATTG-3′, R: 5′-AGATGCTGGGGTCCATGAAAG-3′); Duoxa2 (F: 5′-TAACATTACACTCCGAGGAACAC-3′, R: 5′-AGTCCCTTCTCCAAGGCATG-3′). Housekeeping gene PCR primer sequences for mouse Gusb were (F: 5′-TATGGAGCAGACGCAATCCC-3′, R: 5′-TTCGTCATGAAGTCGGCGAA-3′). Relative expression levels for genes of interest were calculated using the 2^−ddCt^ method. PCR primer sequences used for OTU217 were as follows: F: 5′-TACCGCATAAGCCTGCTGTG-3′, R: 5′-ATCGTTGTCTTGGTAGGCCG-3′. PCR products were resolved by agarose gel electrophoresis.

### 2.6. 16S rRNA Gene Sequencing of Microbiota 

Stool samples from mice were collected and genomic DNA was extracted using the QIAAmp Fast Stool Mini Kit (Qiagen, Redwood City, CA, USA) as per manufacturer instructions. DNA samples were sent to Research and Testing Laboratory (currently RTL Genomics, Lubbock, TX, USA) for processing and analysis based on a previously described protocol [31,32]. 16S ribosomal RNA variable region was amplified and subjected to sequencing on an Illumina MiSeq as previously described [31]. Reads were processed and classified into operational taxonomic units (OTUs) as previously described [32]. Bacterial diversity between groups of mice were compared using the Shannon index (for alpha diversity) and the Bray–Curtis and Jaccard indices (for beta diversity).

### 2.7. Intestinal Organoids 

Organoids were derived from mouse intestines and cultured based on a previously described protocol [33]. Briefly, mouse small intestine was isolated and opened longitudinally. After washing with ice-cold PBS, villi were scraped off using a coverslip. Intestine was then cut into fragments from which crypts were isolated. Crypts were resuspended with Matrigel, and cell suspension plated and cultured to form organoids.

### 2.8. Genomic Analysis 

RNA-seq was performed on mouse intestine and intestinal organoids at the USC Core Lab, based on a previously described protocol [34]. Briefly, the Ovation RNA-Seq System V2 and Ovation Ultralow Library System V2 (NuGEN Technologies, Inc., San Carlos, CA, USA) were used for amplification of total RNA and library preparation. RNA-seq was performed on an Illumina HiSeq 2000 (Illumina, San Diego, CA, USA) using paired-end sequencing. Sequence data were analyzed using Partek Flow software (Partek Inc., Chesterfield, MO, USA). Differentially expressed gene lists were created, and differences were considered significant if false discovery rate (FDR) adjusted *p*-value, i.e., “q-value” < 0.05 or multimodel *p*-value < 0.05. Pathway analysis was performed using Ingenuity Pathway Analysis (IPA) (Qiagen).

### 2.9. Proteomic Analysis 

Samples were prepared and subjected to proteomic analysis by 2DICAL as previously described [9]. Methanol solutions of whole cell extracts were dried and processed for trypsinization. After trypsinization, the obtained peptides were resolved and then quantified. Peptide solution was desalted, dried, and re-dissolved. The obtained peptide solution was subjected to nanoLC-Ultra 2D (AB SCIEX) coupled with to a TripleTOF5600 (AB SCIEX) mass spectrometer. The subjected peptides were directly injected onto a C18, non-end capping, ULTRON HF-ODS(N) (0.1mm I.D., 700 mm length, Shinwa Chemical Industries Ltd., Kyoto, Japan) and then separated by a binary gradient. The masses of the eluted peptides were determined using the TripleTOF5600. MS peaks were detected and quantified using 2DICAL. 2DICAL was developed as a shotgun proteomics analysis system. It analyzes the data of mass to charge ratio (m/z), retention time (RT) and peak intensity generated by liquid chromatography and mass spectrometer (LC/MS), and each sample as elemental data; it deploys various 2-dimensional images with different combinations of axes using these four elements. From the m/z–RT image, peaks derived from the same peptide in the direction of the acquiring time are integrated. By adding algorithms to ensure reproducibility of m/z and RT, the same peak can be compared precisely across different samples, and a statistical comparison of identical peaks in different samples leads to the discovery of specific differentially expressed peptide peaks. Specific peaks are designated by their m/z and RT coordinates, and further analysis is based on these identifiers. The peptide search engine used in 2DICAL is MASCOT software (version 2.5.1; Matrix Science) using the Swiss-Prot mouse database (SwissProt_2016_01.fasta). The mass spectrometry proteomics data have been deposited in the ProteomeXchange Consortium via the jPOST [35] partner repository with the data set identifier PXD021750. Statistical Analyses was performed with the open-source statistical language R (version 3.3.0). The 2DICAL intensity data were converted to protein value averaging the intensity data of peptides derived from the protein.

### 2.10. Ingenuity Pathway Analysis of Proteomic Data 

A total of 2836 proteins were detected in both wild type and p300 S89A mice. The proteomic data were quantile normalized and subjected to differential expression analysis. 93 proteins showed significant changes (fold change (FC) ≤ −1.2 or FC ≥ 1.2, and *p* < 0.05) and were subjected to Ingenuity Pathway Analysis (IPA) (Qiagen, Redwood City, CA, USA) to identify top canonical pathways associated with S89A mutation in mice.

### 2.11. DSS-induced Colitis Mouse Model 

Mice were treated starting on day 1 for up to 5 days with low (2%) dose of dextran sodium sulfate (DSS) administered via the drinking water, based on a previously described protocol [36], after which treatment was discontinued for up to 7 days, and the effect of DSS on % original body weight, histology by hematoxylin and eosin (H&E) and Alcian blue staining, and colon length was assessed.

### 2.12. Data Analysis 

Numerical data were expressed as the means ± standard deviation (s.d.) and Student’s t-test was performed, unless otherwise noted. *p*-values < 0.05 were considered significant.

## 3. Results

### 3.1. Generation of p300 S89A Point Mutant Knock-in Mice

We previously utilized in vitro CRISPR/Cas9 editing of a highly conserved insertion in the N-terminus of p300 (aa 61–70) to demonstrate its importance in regulating Wnt/β-catenin/nuclear receptor interactions [9]. To further explore this critical signaling nexus within the N-terminal domain of the Kat3 coactivator p300, we generated an S89A knock-in point mutation in exon 2 of the mouse p300 gene via site-specific mutagenesis. This mutation removes the highly evolutionarily conserved phosphorylation site at S89 and consequently modulates the interaction of multiple proteins with the N-terminus of p300. The mutant fragment was cloned into a targeting vector for the murine p300 gene. The resultant flip-excision (FLEx) switch construct (Figure S1) was used to generate p300 S89A germ line mice. After crossing the mice with a CMV-Cre mouse line, we confirmed successful introduction of the point mutation and removal of the wild type fragment at both the genomic DNA and messenger RNA levels by PCR and DNA sequencing. The homozygous p300 S89A mice, although born at sub-Mendelian ratios (approximately 50% less than anticipated), did not demonstrate any obvious significant abnormalities (Figure S2 and Table S1) and were fertile, albeit both male and female S89A mice exhibited slightly decreased body weights (<10%) (Figure S2). The mice were subsequently backcrossed with C57BL/6 mice minimally for 10 generations before being used for further experiments.

### 3.2. p300 S89A Mice and Differential β-Catenin Kat3 Coactivator Usage

**Figure 2:**
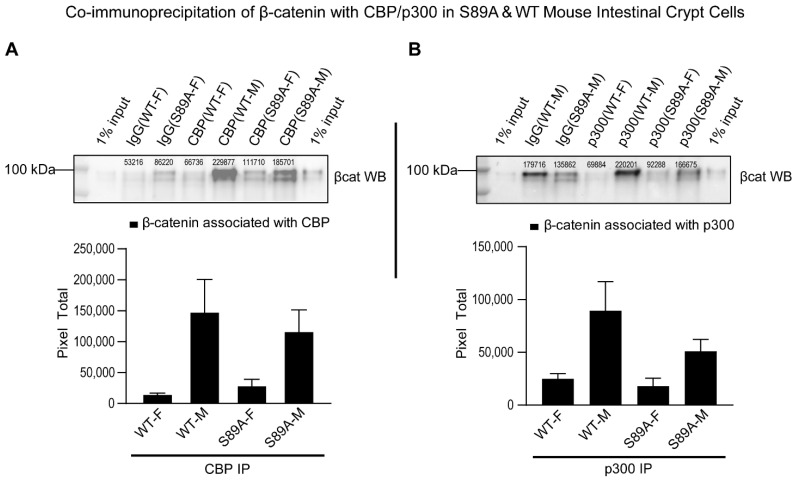
p300 S89A mice show differential usage of Kat3 coactivators CBP and p300 by β-catenin. Co-immunoprecipitation of β-catenin with CBP (**A**) or p300 (**B**) in S89A and WT mouse intestinal crypt cells. Control (IgG) antibody and anti-CBP antibody or anti-p300 antibody were used for immunoprecipitation followed by immunoblotting for β-catenin. Numerical values above protein bands indicate densitometric quantitation of β-catenin associated with CBP or p300. Bar graphs show densitometric quantitation normalized to respective control. Data in bar graphs (mean ± s.e.m.) representative of three independent experiments are shown. S89A, p300 S89A; WT, wild type; F, female; M, male; βcat, β-catenin; IP, immunoprecipitation. Whole immunoblots corresponding to immunoblot data are included in Figure S3.

We previously reported that phosphorylation at S89 of p300 enhanced the association of β-catenin with p300 and mutation of serine 89 to alanine abrogated this phosphorylation dependent increase in vitro [20]. To confirm that this observation was also true in S89A knock-in mice, we performed a co-immunoprecipitation assay using tissue from intestinal crypts in which the Wnt signaling pathway is highly activated. As anticipated based on our previous in vitro studies, the association of β-catenin with p300 was significantly reduced in S89A mice compared with wild type (WT) mice (Figure 2).

### 3.3. p300 S89A Mice Are Extremely Sensitive to Intestinal Insult

**Figure 3:**
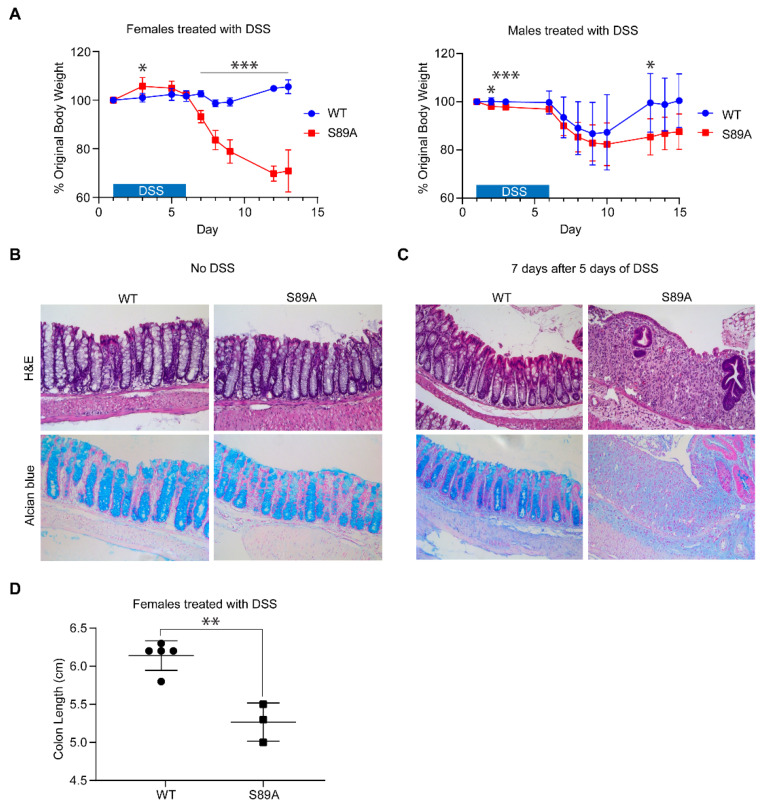
p300 S89A mice are extremely sensitive to intestinal insult. S89A and WT mice were treated starting on day 1 for ~5 days with low (2%) dose of dextran sodium sulfate (DSS) (or vehicle (**B**) after which treatment was discontinued for ~7 days and the effect of DSS on % original body weight (**A**), histology (**B**,**C**), and colon length (**D**) was assessed. Data are mean ± s.d. (*n* = 3–9 per group). * *p* < 0.05, ** *p* < 0.01, *** *p* < 0.001. (**B**) For both WT and S89A mice without DSS treatment: H&E staining shows normal colonic mucosa. The crypt architecture is preserved and there is no evidence of acute, neutrophil-mediated epithelial injury or histologic features suggestive of ongoing, chronic mucosal injury. Alcian blue staining highlights intact goblet cells. (**C**) With DSS treatment: For WT mice: H&E staining shows normal colonic mucosa. The crypt architecture is preserved and there is no evidence of acute, neutrophil-mediated epithelial injury or histologic features suggestive of ongoing, chronic mucosal injury. Alcian blue staining highlights intact goblet cells. For S89A mice: H&E staining shows colonic mucosa with lamina propria replacement by granulation tissue and fibrinopurulent exudate, consistent with ulcer. The few remaining crypts exhibit architectural distortion, indicative of chronic mucosal injury. Alcian blue staining highlights mucin loss and decreased goblet cells, consistent with epithelial injury.

Although p300 S89A mice did not exhibit obvious homeostatic defects under normal feeding and housing conditions, we decided to evaluate their response to insult. Inflammatory bowel disease (IBD), including Crohn’s Disease and ulcerative colitis [36], may arise from infections caused by viruses or bacteria, damage due to ischemia, or disorders of autoimmunity in genetically predisposed individuals. One popular model of colitis utilizes dextran sodium sulfate (DSS) in the drinking water, which damages the intestinal epithelium and a vigorous inflammatory reaction within the intestine generally of several days duration [36]. One variation of this model involves repeated cycles of acute insult with subsequent repair via iterative cycles of DSS administration with intervening periods of recovery, thereby simulating chronic IBD [36]. We chose a relatively low (2%) dose of DSS and proceeded to administer it to seven-week-old female mice, both S89A and wild type C57BL/6 (WT), in their drinking water. After only one round of DSS, effects on control WT mice were minor, with only slight body weight reduction at day eight/nine (~2–3 days after withdrawal of DSS), whereas in sharp contrast, there was a dramatic (~20%) body weight reduction in the mutant mice and two S89A mice died at day 12 (Figure 3A, left panel). A similar trend was observed with male mice (Figure 3A, right panel).

Prior to DSS treatment, the colonic epithelium of S89A mice was normal, essentially the same as WT mice. Prior to treatment, in both S89A and WT mice, crypt architecture was intact and there was no evidence of neutrophil mediated epithelial injury or histological features suggestive of ongoing chronic mucosal injury. Alcian blue staining demonstrated the presence of intact goblet cells (Figure 3B). Histological examination of control and mutant mouse colons at day 12 after DSS treatment (five days treatment, seven days off) showed that S89A mice were nearly devoid of normal colonic epithelium. Hematoxylin and eosin staining showed colonic mucosa with lamina propria replacement by granulated tissue and fibrinopurulent exudate, consistent with ulceration. The few remaining crypts exhibited architectural distortion, indicative of chronic mucosal injury. Alcian blue staining highlighted mucin loss and decreased goblet cells consistent with epithelial injury (Figure 3C), whereas the tissue from WT mice was normal. The colon length of S89A female mice compared to WT controls, one week after 5 days of DSS administration, was somewhat shorter (5.3 versus 6.1 cm) (Figure 3D). 

**Figure 4:**
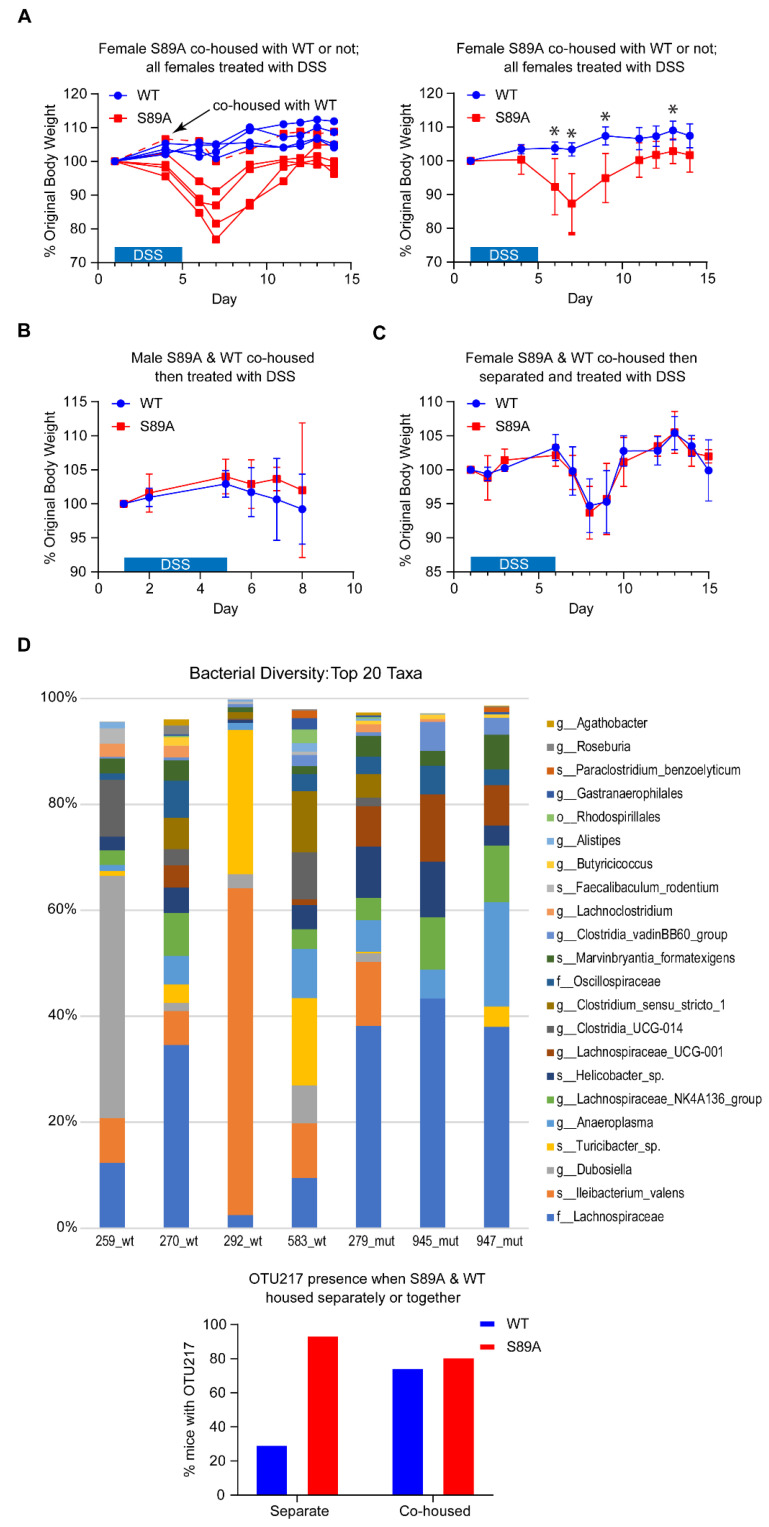
Co-housing of p300 S89A mice with wild type mice modulates severity of intestinal injury potentially via intermixing of microbiota. S89A and WT mice were treated on day 1 for ~4–5 days with low (2%) dose of dextran sodium sulfate (DSS) after which treatment was discontinued for ~7 days (**A**, **C**) or ~2 days (**B**), and the effect of DSS on % original body weight was assessed. A single S89A mouse (dashed red line arrow) which was co-housed with WT mice, from ~4 weeks prior to the start of DSS treatment, was protected from intestinal injury similar to WT mice (**A**, left panel: individual mouse data), while the other S89A mice (not co-housed with WT mice) remained extremely sensitive to intestinal injury (**A**, right panel: aggregate mouse data according to genotype). S89A mice co-housed with WT mice, from ~4 weeks prior to the start of DSS treatment, were protected from intestinal injury (**B**). S89A and wild type mice co-housed ~4 weeks and then separated for ~4 weeks prior to the start of DSS treatment demonstrated an intermediate sensitivity to DSS treatment (**C**). Data are mean ± s.d. (*n* = 4–5 per group) unless otherwise indicated. * *p* < 0.05. (D) 16S rRNA gene sequencing of stool samples from separately housed WT and S89A mice demonstrated large taxonomic differences in the microbiota (**D**, top). (*n* = 3–4 per group.) Bacterial species, OTU-217, was detected by PCR in most of the S89A mice and largely absent in the separately housed WT mice, whereas OTU-217 was detected in most of the co-housed WT and S89A mice (**D**, bottom). (*n* = 12–20 per group.) OTU, operational taxonomic unit.

Perturbations of host–microbiota homeostasis induced by the host genetics and/or environmental factors can fuel inflammation at mucosal surfaces [37,38]. S89A mice were initially housed separately from control mice. We therefore decided to examine whether cohousing and thereby intermixing of the microbiota of S89A and WT mice would affect sensitivity to DSS induced colitis. Although separately housed S89A mice were dramatically more sensitive to 2% DSS treatment (Figure 4A), S89A mice were protected against the effects of the 2% DSS treatment when co-housed with WT mice (Figure 4A, left panel and Figure 4B). Interestingly, if mice subsequently were separated again for four weeks after four weeks of co-housing, both WT and S89A mice had intermediate sensitivity to DSS treatment (Figure 4C). 16S rRNA gene sequencing of stool samples from the separately housed WT and S89A mice demonstrated large taxonomic differences in the microbiota as depicted in a barplot of the top ~20 taxa in each sample (Figure 4D, upper panel). The Shannon (alpha) diversity of the two groups was not significantly different, indicating that the communities had about the same evenness and similar numbers of organisms and similar distribution. On the other hand, the Bray–Curtis and Jaccard (beta) diversity (Figures S5 and S6) was different between the two groups, indicating that the two groups were significantly different in their composition (that is members of the communities). Interestingly, at least one particular bacterial species, OTU-217, which we identified as Kineothrix alysoides and was present in S89A mice yet absent or at very low levels in WT mice housed separately, was transferred effectively during cohousing, and may contribute to enhancing the sensitivity of WT mice to DSS induced colitis (Figure 4D, lower panel). 

### 3.4. Genomic Analysis

**Figure 5:**
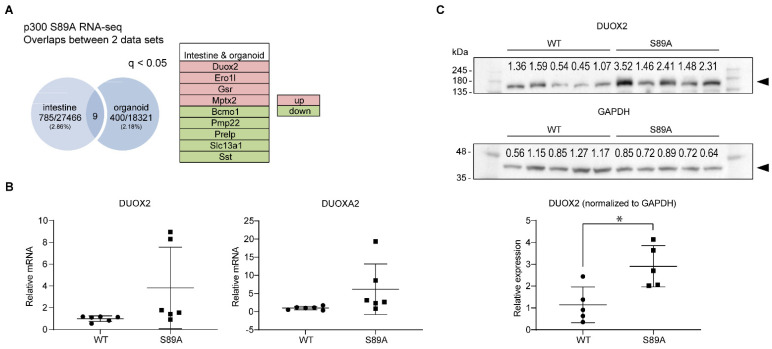
Genomic analysis of p300 S89A mice intestinal tissue. (**A**) RNA-seq analysis identifies 9 genes significantly differentially expressed in intestinal tissue and intestinal organoids derived from S89A mice versus those derived from WT mice. up, up-regulated; down, down-regulated. (*n* = 3 per group.) (**B**) RT-qPCR analysis of Duox2 and Duoxa2 mRNA levels in intestine of S89A and WT mice. (**C**) Immunoblot analysis of Duox2 protein levels in intestinal tissue of S89A and WT mice. Numerical values above protein bands indicate densitometric quantitation. Data in graphs are mean ± s.d. (*n* = 5–6 per group). * *p* < 0.05. Whole immunoblots corresponding to immunoblot data are included in Figure S4.

To further explore the impact of the knock-in point mutation on gene expression, we performed RNA-seq on tissue from the intestines from separately housed untreated p300 S89A and WT mice. To examine epithelium-specific gene expression, intestinal organoids were also grown from both p300 S89A and WT mice and analyzed by RNA-seq. Interestingly, between the organoids and whole ileum RNA-seq (Tables S2 and S3, respectively), there was limited overlap within the statistically significantly regulated genes (400 genes, 2.18% in organoids and 785, 2.86% in ileum q < 0.05), with only nine genes (*DUOX2*, *ERO1l*, *GSR* and *MPTX2* up-regulated and *BCMO1*, *PMP22*, *PRELP*, *SLC13A1* and *SST* down-regulated) (Figure 5A) being common to both. Ingenuity Pathway Analysis (IPA) showed that in the organoids, the top affected network functions were: (1) Embryonic Development, Organismal Development and Function; (2) Cell-To-Cell Signaling and Interaction; (3) Cellular Assembly and Organization, Cell-To-Cell Signaling and Interaction; and (4) Lipid Metabolism, Molecular Transport, Small Molecule Biochemistry. The NRF2-mediated Oxidative Stress Response pathway was also strongly affected. DUOX2 and DUOXA2 members of the NADPH oxidase family, serve as the first line of defense against enteric pathogens by producing microbicidal reactive oxygen species and are the predominant H_2_O_2_-producing system in human colorectal mucosa [39,40]. 

Duox2 expression was significantly increased in both ileum (2.6-fold *p* = 5.00 × 10^−5^) and intestinal organoids (1.5-fold *p* = 7.99 × 10^−6^). We further confirmed increased expression of Duox2 and Duoxa2 at both the message (Figure 5B) and increased expression of Duox2 (~3-fold) at the protein level (Figure 5C) in S89A mice. The transcription factor *GATA4*, which has been demonstrated to play a crucial role in patterning the intestinal epithelium and acts as a critical determinant of enterocyte identity in the jejunum [41] was up-regulated 3.6-fold (*p* = 1.08 × 10^−32^) in intestinal organoids. Among the significantly down-regulated genes in the ileum was *REG3A* (~50% *p* = 5.00 × 10^−5^), an antibacterial C-type lectin, which is constitutively generated in the intestine and displays anti-Gram-positive bactericidal activity [42]. There was also a significant almost 50% reduction in the expression of the interferon-induced transmembrane protein 3, *IFITM3* (*p* = 0.047) in intestinal organoids. Differential expression of *IFITM3* has been found in endoscopic biopsies from Crohn’s Disease patients [43]. In addition, the sulfate transporter *SLC13A1*, an FXR transcriptional target was down-regulated 2.5-fold (*p* = 4.67 × 10^−8^) in organoids with an approximately 50% reduction in ileum (*p* = 5.00 × 10^−5^), consistent with the importance of p300 Ser89 phosphorylation on p300/nuclear receptor interactions [44,45]. Additionally, members of the HOXB cluster, which is critical for specification of the digestive tract [46], including *HOXB3* and *HOXB5-9* were all down-regulated more than 2-fold in intestinal organoids (*p* = 7.97 × 10^−7^ to 4.49 × 10^−5^). Among the top canonical pathways affected in the IPA analysis of the ileum RNA-seq were, interferon signaling, LPS/IL1 mediated inhibition of RXR function, estrogen biosynthesis and fatty acid and xenobiotic metabolism. 1.4-fold increases in Stat1 (*p* = 5.00 × 10^−5^) and Irf8 (*p* = 5.00 × 10^−5^), 2-fold (*p* = 5.00 × 10^−5^) increases in granzyme b (*Gzmb*) and immunity-related GTPase family M member 1 (*Irgm1*) and a 1.6-fold (*p* = 5.00 × 10^−5^) up-regulation of the Interferon Inducible Protein 47 gene (*IFI47*) were observed in S89A mice consistent with the known IFN/STAT1 pathway dysregulation in IBD [47]. Serum Amyloid A1 (*SAA1*), which demonstrates bactericidal action in vitro, may provide a feedback protective mechanism in S89A mice and was increased 5.3-fold (*p* = 5.00 × 10^−5^) [48]. 

### 3.5. Proteomic Analysis

We performed a targeted proteomic analysis of intestinal proteins associated with CBP and/or p300 in wild type versus p300 S89A mice. Interestingly, the aryl hydrocarbon receptor nuclear translocator-like protein 2 (Bmal2) demonstrated increased association with CBP versus p300 in both wild type and p300 S89A mice under both fed or fasted conditions, and although the ratio of CBP to p300 binding did not change significantly in the male mice, fed female p300 S89A mice showed a somewhat decreased Bmal2/CBP versus Bmal2/p300 interaction (Table S4). *BMAL2*, similar to its paralog *BMAL1*, forms a dimer with *CLOCK*, to activate E-box-dependent transcription thereby playing an active role in circadian-regulated transcription [49]. Bmal1 regulates Bmal2, therefore Bmal1 deletion by itself effects combined Bmal1/Bmal2 deletion [50]. Clock/Bmal1-mediated transcription is associated with rhythmic recruitment of Clock to p300 by Bmal1 [51] and differential avidity and timing of binding to CBP versus p300, may affect circadian regulation in S89A mice. Differential association of the N6 methyl adenosine 70 kDa subunit Mettl3 with enhanced p300 association in the female p300 S89A mice was also demonstrated. These results are interesting given the recent report of circadian clock regulation of lipid metabolism and in particular PPARα-mediated transcription being modulated by m6A mRNA methylation [52] and the role of p300 Ser89 in PPAR transcriptional regulation [23]. The effect of the p300 S89A mutation on circadian regulation was not investigated in this study, however given the effect of this mutation on nuclear receptor signaling in both the intestine and in the liver (to be reported separately), and the crosstalk between nuclear receptors and core circadian transcriptional regulators [53], this area will be the focus of future investigations.

**Figure 6:**
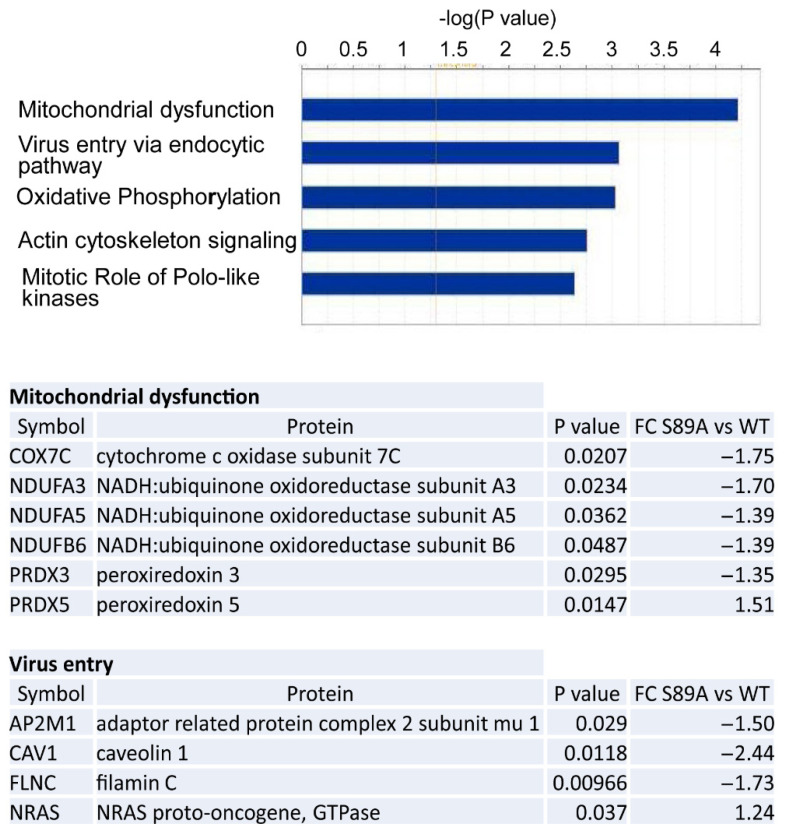
Bioinformatic analysis of proteins differentially expressed in p300 S89A mice intestinal tissue. IPA bioinformatic analysis of the 93 genes identified by proteomic analysis to be significantly differentially expressed in intestinal tissues of S89A versus WT mice (with fold change ≤ −1.2 or fold change ≥ 1.2 and *p* < 0.05) revealed the Mitochondrial dysfunction and Virus entry via endocytic pathway as top canonical pathways associated with S89A mutation (top). Differentially expressed proteins comprising the Mitochondrial dysfunction and Virus entry via endocytic pathways and associated fold change (FC) in intestinal tissues of S89A versus WT mice (bottom). (*n* = 3–4 per group).

We next undertook global proteomic analysis of intestinal tissue from wild type and p300 S89A mice (Table S5), which revealed that 93 of the 2836 proteins detected in both WT and S89 mice proteins were significantly differentially expressed (with fold change ≤ −1.2 or fold change ≥ 1.2 and *p* < 0.05) (Table S6). IPA analysis of the 93 proteins showed that Mitochondrial Dysfunction, Oxidative Phosphorylation and Virus Entry via Endocytic Pathway were the top canonical pathways associated with S89A mutation (Figure 6). Interestingly, we have identified similar effects on mitochondrial dysfunction and oxidative phosphorylation in other organ systems in S89A mice (e.g., brain, liver, adipose tissue, to be reported separately) as well as cell-based model systems that affect differential Kat3 coactivator usage (i.e., P19 p300 N-terminally edited cells Ono et al. [9]). Metabolic dysfunction appears to be a fundamental feature associated with aberrant differential Kat3/β-catenin coactivator usage (Kahn lab manuscript in preparation). Further, the protein expression level of the bile acid transporter protein FABP6, the 2nd most down-regulated protein found in S89A mice, was approximately 10% of that in their wild type counterparts. This result is consistent with FABP6, which is required for efficient absorption and transport of bile acids in the distal intestine, being a PPAR target gene [54] that is repressed by GATA4 in the small intestine. *FABP6* message was also significantly decreased in S89A mice (*p* = 0.00035). Bile acids and their FXR nuclear receptors play important roles in inflammatory response and intestinal barrier function and are involved in IBD pathophysiology [55]. Calreticulin (CALR), which appears to play a role in leukocyte infiltration in mouse models of colitis via its interaction with alpha integrins, was down-regulated in S89A mice (~30%) [56]. Calreticulin, also is secreted by macrophages and binds to target cells marking them for removal by programmed cell phagocytosis [57] and believed to function as an “eat me” signal. Viable cells also can expose calreticulin on their surfaces, apparently protected from engulfment via concurrently expressed so called “don’t eat me” signals, e.g., CD200 and CD47 [57]. Interestingly, the expression of the OX-2 membrane glycoprotein (CD200) is increased 1.5-fold in S89A intestines. The role of these differentially expressed proteins in innate immunity and the intestinal phenotype displayed in S89A mice will require further investigation. 

### 3.6. p300 S89A Is a Part of a 14-3-3 Binding Motif

**Figure 7:**
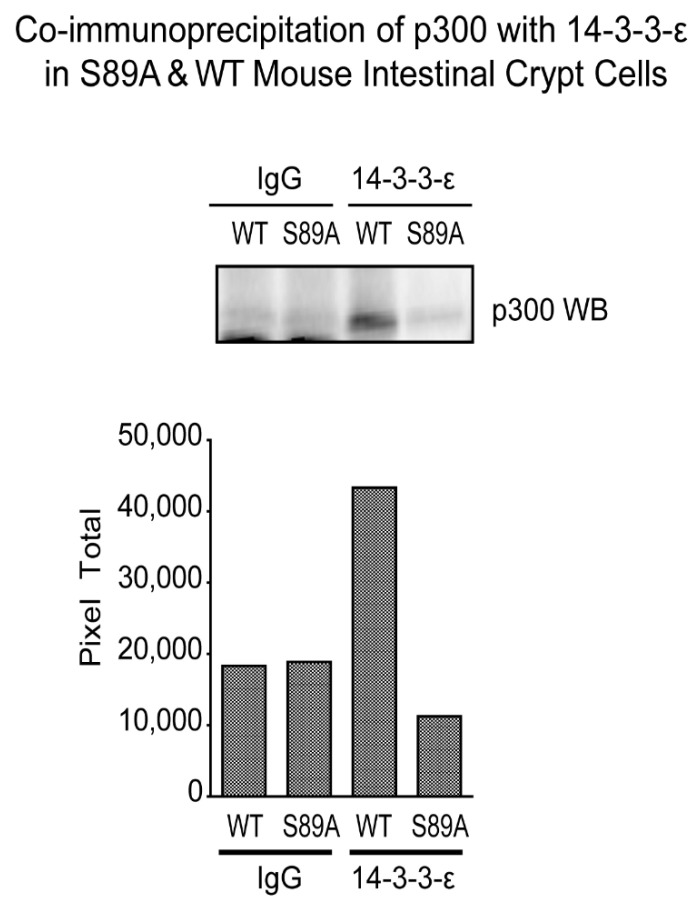
Association of 14-3-3ε with p300 is decreased in intestinal tissue from p300 S89A mice. Co-immunoprecipitation of p300 with 14-3-3ε in S89A and WT mouse intestinal crypt cells. Control (IgG) antibody and anti-14-3-3ε antibody were used for immunoprecipitation followed by immunoblot for p300. Bar graphs show densitometric quantitation of p300 associated with 14-3-3ε versus control IgG in intestinal tissues of S89A versus WT mice.

The 14-3-3 protein family of scaffolding chaperones regulates diverse intracellular signaling pathways [58]. We observed that the p300 sequence LLRSGSSP (aa 84–91) is a member of the consensus 14-3-3 binding site sequence (LX(R/K)SX(pS/pT)XP) [59]. It is unique to p300 and not conserved in CBP. We therefore anticipated that mutation of serine 89 to alanine would disrupt the binding of 14-3-3 proteins to p300. Immunoprecipitation of 14-3-3 epsilon (14-3-3ε) was performed using protein from intestines of WT and S89A mice and subsequently immunoblotted with an antibody specific for p300. As shown (Figure 7) a substantial decrease in the association of 14-3-3ε with p300 was demonstrated in intestinal tissue from S89A mutant mice. To confirm these findings, we carried out the reverse experiment, i.e., anti-p300 antibody was used for immunoprecipitation followed by immunoblotting for 14-3-3ε. Again, we found a substantial decrease in the association of 14-3-3ε with p300 in S89A mutant mouse intestinal tissue (Figure S7). Further studies are needed to address the importance of this interaction, however differential subcellular localization of p300 regulated by its interaction with 14-3-3 proteins could potentially affect its role as a nuclear transcriptional coactivator.

## 4. Discussion

Differential Kat3 coactivator usage by β-catenin is a fundamental regulatory mechanism in stem cell maintenance and the initiation of differentiation and repair [1,60]. Stem cells in their respective niches receive a myriad of information including oxygen and nutrient levels, circadian input, adhesion molecules, cell–cell contacts, growth factors, etc., to decide to maintain quiescence or to enter the cell cycle and undergo either symmetric or asymmetric division [1,61]. The extreme N-terminal 111 amino acids of CBP and p300, decidedly the most divergent regions of the two Kat3 coactivators [2], contain binding domains for β-catenin, nuclear receptors [9] and Stat1, an interferon-dependent transcription factor [62], as well as approximately 20 serine/threonine residues [1,2,63,64]. Post-translational modifications of these serine/threonine residues (by phosphorylation or dephosphorylation) [21,22,23,24] and combinatorial interactions, both antagonistic and synergistic [9], of multiple transcription factor families (i.e., β-catenin/TCF/LEF, β-catenin/FOXO, nuclear receptors, e.g., RAR, VDR, PPAR, etc., Stat1, 2 as well as others), provide a unique mechanism to integrate a diverse array of signal inputs. We previously demonstrated that a highly evolutionarily conserved 27bp/9aa insertion in the N-terminus of p300, which is not present in CBP, between the conserved β-catenin-binding region (DELI-sequence) and the nuclear receptor binding sequence (LXXLL), determines if the interaction will be potentially synergistic or purely antagonistic between the Wnt/β-catenin and nuclear receptor signaling cascades [2,9]. 

To further investigate mechanisms of signal integration effected by this domain of the Kat3 family, we generated p300 S89A knock-in mice. Based upon our earlier pharmacologic studies, p300 S89 is critical for controlling differential coactivator usage by β-catenin via post-translational phosphorylation in stem/progenitor populations [1,20,26] and Serine 89 appears to be a target for a number of kinase cascades [21,22,23,24]. Although, the p300 S89A polymorphism has not been reported in humans, to the best of our knowledge, homozygous p300 S89A mice, albeit born at sub-Mendelian ratios and exhibiting slightly decreased body weights, were relatively normal and fertile. However, after insult/stress and with aging (to be published separately later), we have found a range of interesting phenotypes associated with this single point mutation. Herein we report our initial findings regarding the intestinal phenotype of the p300 S89A mice. We first investigated the interaction of β-catenin with p300 in intestinal crypts, a region associated with activated Wnt signaling and found as anticipated that it was significantly reduced in S89A mice (Figure 2, right panel). Decreased β-catenin/p300 interaction in the p300 S89A mice did not manifest itself in obvious defects in either ileal or colonic architecture under normal homeostatic conditions (Figure 3C). However, treatment with a mild insult (2% DSS), while having minimal effects on wild type C57BL/6 mice, had a striking effect on the p300 S89A mice as evidenced by the development in the p300 S89A mice of severe colitis, a significant risk factor predisposing to colorectal cancer [27,28] (Figure 3A,B). 

Further investigation demonstrated cell intrinsic differences in the intestinal epithelium, based upon RNA-seq of intestinal organoids, as well as microbiome compositional differences and differential immune responses in the intestine. Interestingly, S89A mice separately housed were dramatically more sensitive (Figure 4A) than when co-housed with WT mice (Figure 4A, left panel and Figure 4B). However, when separated again for four weeks after being co-housed for four weeks, S89A mice demonstrated intermediate sensitivity to DSS treatment (Figure 4C). These results point to a complex interplay between host intrinsic differences in the epithelium and extrinsic interaction with the intestinal microbiome associated with differential microbiome colonization and metabolite production [65,66] the host immune response, both innate and adaptive [67], related to a single amino acid variance within the highly conserved and critical region of signal integration in p300 [1,68]. 

Global genomic and proteomic analysis showed a number of prominent pathway differences including lipid metabolism, oxidative stress response, mitochondrial function and oxidative phosphorylation. We have found in further analyses of other organ systems (liver, brain and adipose tissue) that these are fundamental differences generally associated with differential Kat3/β-catenin coactivator usage. Notably, sulfate transporter *SLC13A1*, an FXR transcriptional target was significantly down-regulated in both organoids and ileum of S89A mice, consistent with the importance of p300 Ser89 phosphorylation on p300/nuclear receptor interactions [44,45]. Sulfate insufficiency impedes detoxification, heightens the risk of xenobiotic toxicity, and modifies the activity and metabolism of numerous physiologic compounds, including proteoglycans, hormones, and neurotransmitters [69] and very recently integrated microbiota and metabolite profiles linked Crohn’s disease with sulfur metabolism [65]. Interestingly, the levels of both *p*-cresol sulfate and phenol sulfate, potentially toxic intestinal bacterial fermentation products [70], were significantly upregulated in the liver metabolome of S89A mice (to be published separately). Given the importance of cellular metabolism and mitochondrial function in the regulation of the immune response [71,72], further investigations with regard to intestinal immunity in the p300 S89A mice and the effects in other organ systems are ongoing and will be reported in due course. Additionally, mitochondrial activity has been linked to maintaining a state of physiological hypoxia at the colonic surface. Limiting the amount of oxygen at the mucosal surface controls the aerobic growth of facultative anaerobic bacteria [73], whereas reduced mitochondrial bioenergetics decreases epithelial oxygen consumption, thereby increasing epithelial oxygenation and the diffusion of oxygen into the intestinal lumen [74,75,76]. Recent experimental evidence [77] has provided support for the hypothesis that an expansion of facultative anaerobic bacteria in IBD patients are secondary to changes in epithelial energy metabolism [78]. Furthermore, it was demonstrated that treating mitochondrial dysfunction in the colon using the PPAR agonist 5-ASA [79], consistent with the dysfunctional PPAR signaling associated with S89A mutation, ameliorated signs of disease in mice with pre-IBD and normalized the microbiota composition by restoring epithelial hypoxia [77]. 

The diverse array of effects on fundamental processes including epithelial differentiation, metabolism, immune response and microbiome colonization, all brought about by a single amino acid modification S89A, highlights the role of this region in the Kat3 coactivator p300 as a critical signaling nexus and the rationale for conservation of this residue and surrounding region for hundreds of million years of vertebrate evolution. Additional studies related to the fundamental regulation of metabolism via differential Kat3/β-catenin usage and its roles in development and disease will be reported in due course.

## 5. Conclusions

We describe the generation of novel p300 S89A knock-in mice carrying a single site directed amino acid mutation in p300, changing the highly evolutionarily conserved serine 89 to alanine, thus enhancing Wnt/CBP/catenin signaling (at the expense of Wnt/p300/catenin signaling). We show that S89A mice are extremely sensitive to intestinal insult resulting in colitis, which is known to significantly predispose to colorectal cancer. We demonstrate cell intrinsic differences, and microbiome compositional differences and differential immune responses, in the intestines of S89A versus wild type mice. Genomic and proteomic analyses reveal pathway differences, including lipid metabolism, oxidative stress response, mitochondrial function and oxidative phosphorylation. The diverse effects on fundamental processes including epithelial differentiation, metabolism, immune response and microbiome colonization, all brought about by a single amino acid modification S89A, highlights the critical role of this region in p300 as a signaling nexus in development and disease (e.g., inflammation and cancer) and the rationale for conservation of this residue and surrounding region for hundreds of million years of vertebrate evolution.

## Data Availability

Proteomics data have been deposited in the ProteomeXchange Consortium via the jPOST [35] partner repository with the data set identifier PXD021750. Additional genomics and proteomics data have been included in Supplementary Materials. All other data supporting the findings of this study are available from the corresponding author on reasonable request.

## Supplementary Materials

The following are available online at https://www.mdpi.com/2072-6694/13/6/1288/s1, Figure S1: Schematic of flip-excision switch construct used to create p300 S89A germ line mice, Figure S2: Body weight and blood count did not show any major abnormalities between p300 S89A mice and wild type mice, Figure S3: p300 S89A mice show differential usage of Kat3 coactivators CBP and p300 by β-catenin, Figure S4: Immunoblot analysis of Duox2 protein levels in intestinal tissue of S89A and WT mice, Figure S5: Bray–Curtis (beta) diversity between wild type and p300 S89A mutant mouse groups, Figure S6: Jaccard (beta) diversity between wild type and p300 S89A mutant mouse groups, Figure S7: Association of 14-3-3ε with p300 is decreased in intestinal tissue from p300 S89A mice, Table S1: Comparison of Blood Chemistry between p300 S89A and Wild Type Mice, Table S2: Organoids RNA-seq, Table S3: Intestine RNA-seq, Table S4: Intestine co-IP of CBP vs. p300 proteomics, Table S5: Intestine global proteomics, Table S6: Proteomic Identification of Differentially Expressed Proteins in p300 S89A vs. Wild Type Mice.
