# Supplementary material for: p300 Serine 89: A Critical Signaling Integrator and Its Effects on Intestinal Homeostasis and Repair
Source: Cancers (Basel). 2021 Mar 14;13(6):1288. doi: 10.3390/cancers13061288 (PMC7999107; doi:10.3390/cancers13061288)
Supplement: Supplementary file 1 [file cancers-13-01288-s001.zip › cancers-1116787-supplementary/cancers-1116787-supp.docx]

Supplementary Materials: p300 Serine 89: A Critical Signaling Integrator and Its Effects on Intestinal Homeostasis and Repair

Keane K. Y. Lai, Xiaohui Hu, Keisuke Chosa, Cu Nguyen, David P. Lin, Keith K. Lai, Nobuo Kato, Yusuke Higuchi, Sarah K. Highlander, Elizabeth Melendez, Yoshihiro Eriguchi, Patrick T. Fueger, Andre J. Ouellette, Nyam-Osor Chimge, Masaya Ono and Michael Kahn


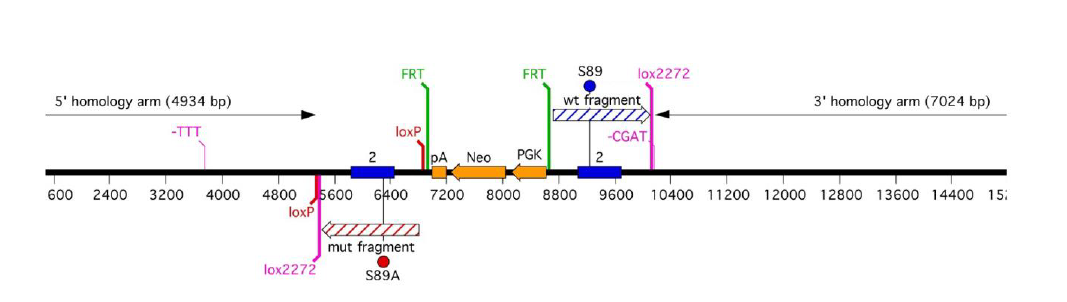


**Figure S1.** Schematic of flip-excision switch construct used to create p300 S89A germ line mice. The S89A knock-in point mutation in exon 2 of the mouse p300 gene (via site-specific mutagenesis) was generated using the flip-excision (FLEx) switch construct depicted. This mutation removes the highly conserved phosphorylation site at S89. The construct included five segments: the 5’ homology arm, a point-mutated exon 2 in inverted orientation, a PGK-Neo selection cassette, the wild type exon 2, and the 3’ homology arm. In principle, transcription from the mutant exon 2 should have been activated via Cre recombinase in two steps. First, Cre recombinase would invert the mutated fragment flanked by the loxP site to correct the orientation to activate, and then excise the wild type fragment flanked by lox2272 to inactivate it. However, we found that mice homozygous for the knock-in construct displayed early embryonic stage lethality, similar to that of p300 knockout mice, suggesting that the wild type p300 protein was not being produced properly from the wild type fragment in vivo for unknown reasons. This malfunction of the wild type fragment caused us to revise our original plan of conditional mutagenesis, resulting in our decision to generate p300 S89A germ line mice.


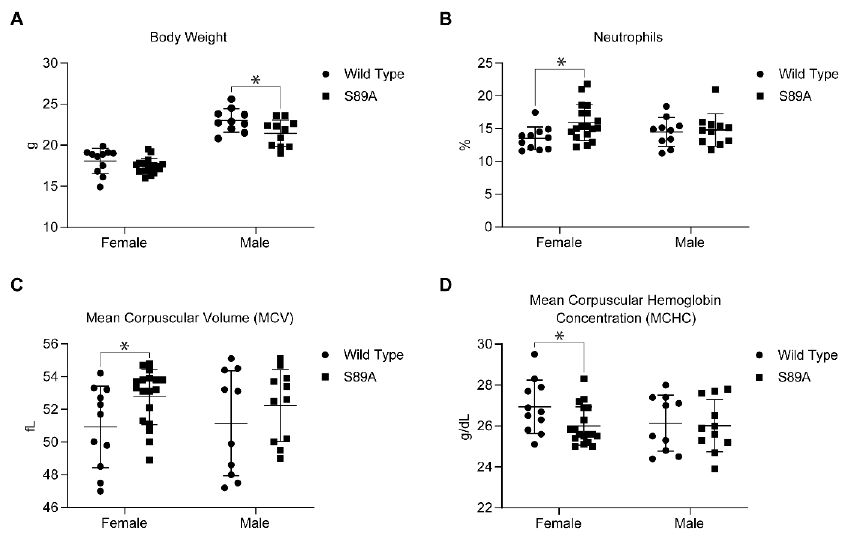


**Figure S2.** Body weight and blood count did not show any major abnormalities between p300 S89A mice and wild type mice. Comparison of means between gender-matched 7–8 week-old p300 S89A mice and wild type mice for body weight (**A**), percent neutrophils (**B**), mean corpuscular volume (**C**), and mean corpuscular hemoglobin concentration (**D**). Data in graphs are mean ± s.d. (*n* = 10–18 per group). **P* < 0.05.


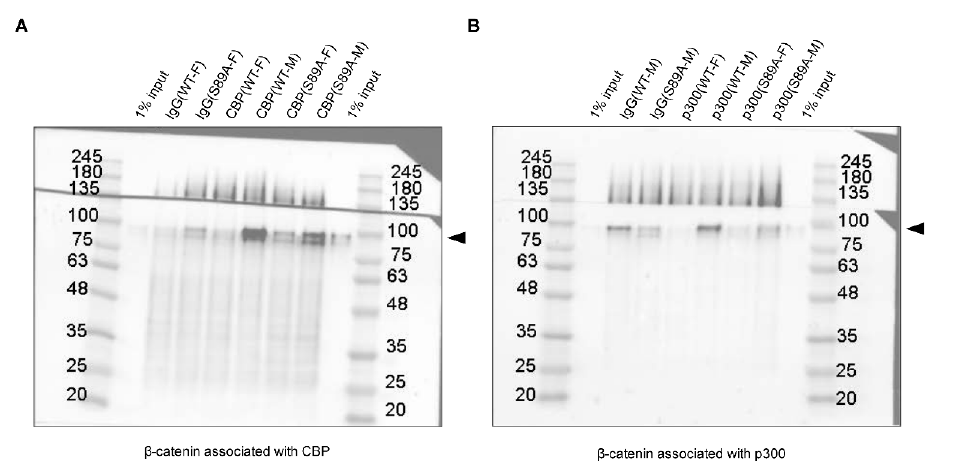


**Figure S3.** p300 S89A mice show differential usage of Kat3 coactivators CBP and p300 by β-catenin. Whole immunoblots corresponding to immunoblot data presented in Figure 2. Co-immunoprecipitation of β-catenin with CBP (**A**) or p300 (**B**) in S89A and WT mouse intestinal crypt cells. Control (IgG) antibody and anti-CBP antibody or anti-p300 antibody were used for immunoprecipitation followed by immunoblot for β-catenin (as indicated by arrows). S89A, p300 S89A; WT, wild type; F, female; M, male. Numerical values adjacent to markers indicate molecular weight in kDa.


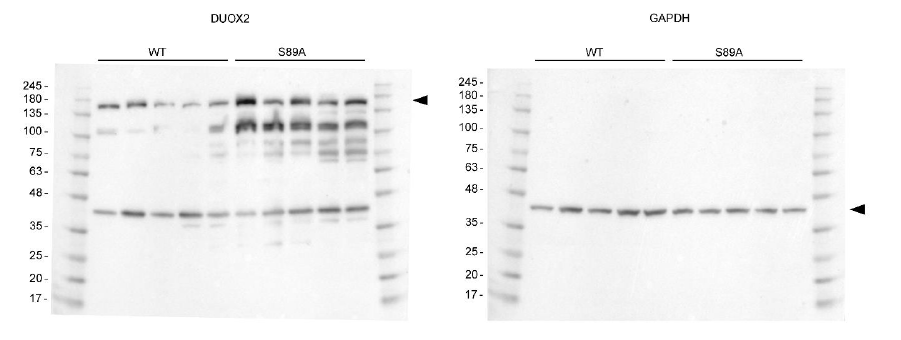


**Figure S4.** Immunoblot analysis of Duox2 protein levels in intestinal tissue of S89A and WT mice. Whole immunoblots corresponding to data presented in Figure 5C. Immunoblot for Duox2 or control Gapdh (as indicated by arrows). S89A, p300 S89A; WT, wild type. Numerical values adjacent to markers indicate molecular weight in kDa.


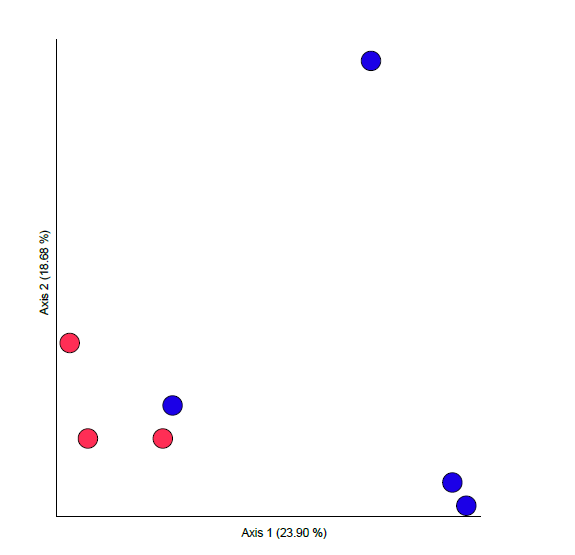


**Figure S5.** Bray-Curtis (beta) diversity between the wild type (blue) and p300 S89A mutant (red) mouse groups, indicating that the two groups were significantly different in their composition (that is members of the communities).


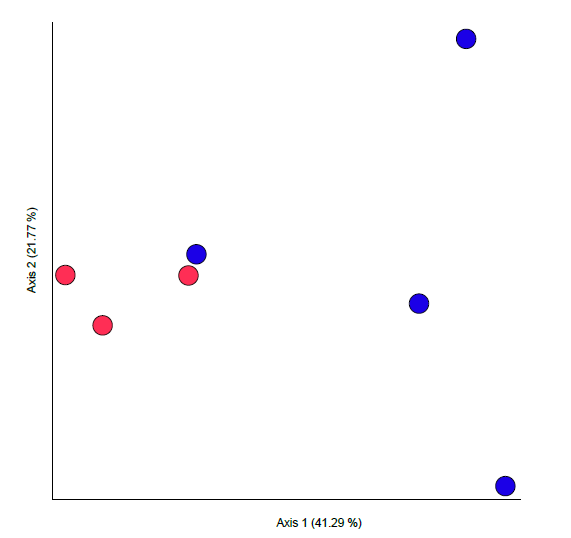


**Figure S6.** Jaccard (beta) diversity between the wild type (blue) and p300 S89A mutant (red) mouse groups, indicating that the two groups were significantly different in their composition (that is members of the communities).


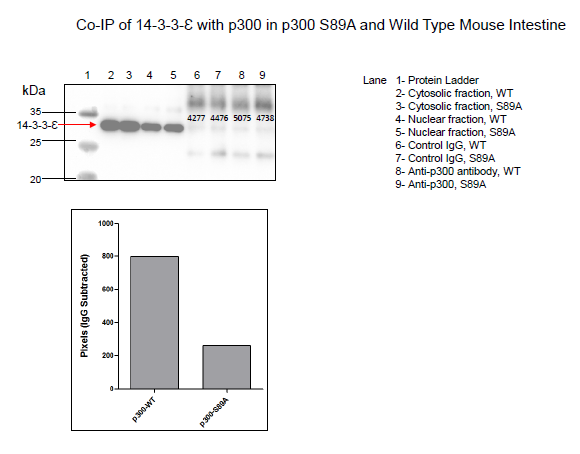


**Figure S7.** Association of 14-3-3ε with p300 is decreased in intestinal tissue from p300 S89A mice. Co-immunoprecipitation of 14-3-3ε with p300 in S89A and WT mouse intestinal cells. Control (IgG) antibody and anti-p300 antibody were used for immunoprecipitation followed by immunoblot for 14-3-3ε. Numerical values above protein bands indicate densitometric quantitation of 14-3-3εassociated with p300 (or control IgG). Bar graphs show densitometric quantitation of 14-3-3ε associated with p300 normalized to control IgG in intestinal tissues of S89A versus WT mice.

**Table S1.** Comparison of Blood Chemistry between p300 S89A and Wild Type Mice.

| **Chemistry Test** | **P value (from t-test)** |
| --- | --- |
| Total Protein | 0.744 |
| Albumin | 0.736 |
| Globulin | 0.848 |
| Albumin/Globulin Ratio | 0.908 |
| Aspartate Aminotransferase | 0.075 |
| Alanine Aminotransferase | 0.854 |
| Alkaline phosphatase | 0.668 |
| Gamma-glutamyl transferase | 0.169 |
| Total Bilirubin | 0.868 |
| Blood Urea Nitrogen (BUN) | 0.655 |
| Creatinine | 1.000 |
| BUN/Creatinine Ratio | 0.655 |
| Phosphorus | 0.543 |
| Glucose | 0.212 |
| Calcium | 0.362 |
| Magnesium | 0.508 |
| Sodium (Na) | 0.303 |
| Potassium (K) | 0.198 |
| Na/K Ratio | 0.259 |
| Chloride | 0.702 |
| Cholesterol | 0.695 |
| Triglyceride | 0.765 |
| Amylase | 0.728 |
| Lipase | 0.242 |
| Creatine Phosphokinase | 0.122 |

*n* = 8-9 per group. Clinical chemistry testing as listed in the Table was performed on blood from p300 S89A and wild type mice. Mean values per test for p300 S89A versus wild type mice were compared using the Student’s t-test.

**Table S2.** Organoids RNA-seq.

**Table S3.** Intestine RNA-seq.

**Table S4.** Intestine co-IP of CBP vs. p300 proteomics.

**Table S5.** Intestine global proteomics.

**Table S6.** Proteomic Identification of Differentially Expressed Proteins in p300 S89A vs. Wild Type Mice.

| **Symbol** | **Protein** | **P value**  **(S89A vs WT)** | **Fold Change**  **(S89A vs WT)** |
| --- | --- | --- | --- |
| PRAF3 | PRA1 family protein 3 | 0.00918214 | 2.58 |
| MYG | Myoglobin | 0.00225183 | 2.48 |
| PCLO | Protein piccolo | 0.00956903 | 2.01 |
| DDX21 | Nucleolar RNA helicase 2 | 0.0284004 | 1.95 |
| SGTB | Small glutamine-rich tetratricopeptide repeat-containing protein beta | 0.033929 | 1.91 |
| BCAS1 | Breast carcinoma-amplified sequence 1 homolog | 0.0459885 | 1.87 |
| SGPL1 | Sphingosine-1-phosphate lyase 1 | 0.0450239 | 1.87 |
| EFG1 | Elongation factor G 1, mitochondrial | 0.0269061 | 1.64 |
| ABHEB | Abhydrolase domain-containing protein 14B | 0.0126882 | 1.62 |
| PGES2 | Prostaglandin E synthase 2 | 0.0205592 | 1.61 |
| HAOX2 | Hydroxyacid oxidase 2 | 0.0444709 | 1.56 |
| GSDMD | Gasdermin-D | 0.046353 | 1.54 |
| AAK1 | AP2-associated protein kinase 1 | 0.0159375 | 1.52 |
| PRDX5 | Peroxiredoxin-5, mitochondrial | 0.0146951 | 1.51 |
| CAB39 | Calcium-binding protein | 0.0111922 | 1.50 |
| NPL | N-acetylneuraminate lyase | 0.02225 | 1.49 |
| PHAR1 | Phosphatase and actin regulator 1 | 0.0402639 | 1.45 |
| MLRS | Myosin regulatory light chain 2, skeletal muscle isoform | 0.0237246 | 1.42 |
| CPLX1 | Complexin-1 | 0.0108965 | 1.41 |
| ABLM2 | Actin-binding LIM protein 2 | 0.0467177 | 1.40 |
| UGDH | UDP-glucose 6-dehydrogenase | 0.016522 | 1.38 |
| RS14 | 40S ribosomal protein S14 | 0.0117811 | 1.38 |
| KCRU | Creatine kinase, ubiquitous mitochondrial | 0.0463191 | 1.38 |
| SV2B | Synaptic vesicle glycoprotein 2B | 0.045272 | 1.37 |
| PI51A | Phosphatidylinositol-4-phosphate 5−kinase type-1 alpha | 0.0303205 | 1.36 |
| PI51C | Phosphatidylinositol-4-phosphate 5−kinase type-1 gamma | 0.0303205 | 1.36 |
| PGRC2 | Membrane−associated progesterone receptor component 2 | 0.00350162 | 1.35 |
| AAAD | Arylacetamide deacetylase | 0.0379928 | 1.35 |
| EHD3 | EH domain-containing protein 3 | 0.0374782 | 1.34 |
| HXK4 | Glucokinase | 0.0309104 | 1.34 |
| DDI2 | Protein DDI1 homolog 2 | 0.0464067 | 1.28 |
| VPS52 | Vacuolar protein sorting-associated protein 52 homolog | 0.00840107 | 1.27 |
| GTR2 | Solute carrier family 2, facilitated glucose transporter member 2 | 0.023336 | 1.27 |
| H17B6 | Hydroxysteroid 17-beta dehydrogenase 6 | 0.0369817 | 1.26 |
| S6A19 | Sodium-dependent neutral amino acid transporter B(0) | 0.02542 | 1.25 |
| RL38 | 60S ribosomal protein L38 | 0.0353214 | 1.24 |
| SFXN1 | Sideroflexin-1 | 0.0124022 | 1.24 |
| RASN | GTPase Nras | 0.0369695 | 1.24 |
| 2A5E | Serine/threonine-protein phosphatase 2A 56 kDa regulatory subunit epsilon isoform | 0.0142328 | 1.23 |
| SCOT1 | Succinyl-CoA:3-ketoacid-coenzyme A transferase 1, mitochondrial | 0.00529715 | −1.23 |
| CP4AE | Cytochrome P450 4A14 | 0.0113723 | −1.23 |
| PACN1 | Protein kinase C and casein kinase substrate in neurons protein 1 | 0.0218851 | −1.24 |
| TMED5 | Transmembrane emp24 domain−containing protein 5 | 0.0132731 | −1.25 |
| PIMT | Protein-L-isoaspartate(D-aspartate) O-methyltransferase | 0.0112384 | −1.28 |
| PTPA | Serine/threonine-protein phosphatase 2A regulatory subunit B~ | 0.000725532 | −1.29 |
| TLN1 | Talin−1 | 0.0138377 | −1.31 |
| PRDX3 | Thioredoxin-dependent peroxide reductase, mitochondrial | 0.0294819 | −1.35 |
| NP1L1 | Nucleosome assembly protein 1-like 1 | 0.0383379 | −1.36 |
| EMB | Embigin | 0.0109525 | −1.37 |
| NDUB6 | NADH dehydrogenase [ubiquinone] 1 beta subcomplex subunit 6 | 0.048707 | −1.39 |
| NDUA5 | NADH dehydrogenase [ubiquinone] 1 alpha subcomplex subunit 5 | 0.0361821 | −1.39 |
| GD1L1 | Ganglioside-induced differentiation-associated protein 1−like 1 | 0.0201876 | −1.40 |
| RBM3 | Putative RNA-binding protein 3 | 0.00357973 | −1.45 |
| LCAP | Leucyl-cystinyl aminopeptidase | 0.0383192 | −1.46 |
| MIF | Macrophage migration inhibitory factor | 0.0260791 | −1.46 |
| TSR2 | Pre-rRNA-processing protein TSR2 homolog | 0.0286351 | −1.47 |
| CYGB | Cytoglobin | 0.00196232 | −1.49 |
| AP2M1 | AP-2 complex subunit mu-1 | 0.0290121 | −1.50 |
| PTAD1 | Protein tyrosine phosphatase−like protein PTPLAD1 | 0.0133349 | −1.52 |
| PUR6 | Multifunctional protein ADE2 | 0.0410255 | −1.53 |
| PDC6I | Programmed cell death 6-interacting protein | 0.0407175 | −1.54 |
| RAB35 | Ras−related protein Rab-35 | 0.0354715 | −1.57 |
| TGFI1 | Transforming growth factor beta-1-induced transcript 1 protein | 0.049051 | −1.59 |
| COTL1 | Coactosin-like protein | 0.0274769 | −1.62 |
| FIBB | Fibrinogen beta chain | 0.0142941 | −1.65 |
| SAE2 | SUMO-activating enzyme subunit 2 | 0.0180463 | −1.67 |
| SYNEM | Synemin | 0.00259576 | −1.69 |
| RIFK | Riboflavin kinase | 0.0296825 | −1.70 |
| NDUA3 | NADH dehydrogenase [ubiquinone] 1 alpha subcomplex subunit 3 | 0.0234492 | −1.70 |
| ANXA1 | Annexin A1 | 0.0209203 | −1.71 |
| FLNC | Filamin-C | 0.00965532 | −1.73 |
| COX7C | Cytochrome c oxidase subunit 7C, mitochondrial | 0.0207211 | −1.75 |
| RAB10 | Ras−related protein Rab-10 | 0.0256923 | −1.84 |
| DHSO | Sorbitol dehydrogenase | 0.0151407 | −1.89 |
| GPM6A | Neuronal membrane glycoprotein M6-a | 0.0202245 | −1.94 |
| LEG1 | Galectin-1 | 0.0353706 | −2.02 |
| GBRA1 | Gamma−aminobutyric acid receptor subunit alpha-1 | 0.0497253 | −2.14 |
| PSB9 | Proteasome subunit beta type-9 | 0.0287108 | −2.16 |
| CIRBP | Cold−inducible RNA−binding protein | 0.00205451 | −2.18 |
| PSB1 | Proteasome subunit beta type-1 | 0.017556 | −2.18 |
| RM21 | 39S ribosomal protein L21, mitochondrial | 0.00152292 | −2.20 |
| EVC | Ellis−van Creveld syndrome protein homolog | 0.0318199 | −2.29 |
| HA2B | H−2 class II histocompatibility antigen, A-B alpha chain | 0.0466119 | −2.37 |
| HA2U | H−2 class II histocompatibility antigen, A-U alpha chain (Fragment) | 0.0466119 | −2.37 |
| PLK3 | Serine/threonine-protein kinase PLK3 | 0.00180448 | −2.41 |
| CAV1 | Caveolin-1 | 0.0118325 | −2.44 |
| FSIP1 | Fibrous sheath-interacting protein 1 | 0.0115349 | −2.51 |
| CK067 | UPF0366 protein C11orf67 homolog | 0.0231243 | −2.85 |
| CP035 | UPF0171 protein C16orf35 homolog | 0.00998893 | −3.28 |
| S10A6 | Protein S100-A6 | 0.00704094 | −3.58 |
| SCRN1 | Secernin-1 | 0.0268366 | −4.07 |
| IGKC | Ig kappa chain C region | 0.0370728 | −6.17 |
| EIF3L | Eukaryotic translation initiation factor 3 subunit L | 0.00462065 | −6.99 |

93 proteins identified by proteomic analysis to be significantly differentially expressed in intestinal tissues of p300 S89A versus wild type mice with fold change ≤ -1.2 or fold change ≥ 1.2 and P < 0.05. (n = 3-4 per group.)
